# Welding of Gold

**Published:** 1888-03

**Authors:** 


### WELDING OF GOLD.

A valued correspondent propounds a series of questions of which the following is a summary :

"What would be the difference in unit pounds—if any—necessary to a perfect weld of two pieces of soft iron at a white heat, between strikes of a hammer and ponderous pressure without concussion ?"

The queries refer to the rotary *versus* the mallet system of filling teeth, and the natural inference to be drawn from them is, that as iron welds more readily and perfectly under blows from a hammer than from mere pressure, the same rule will hold good in consolidating a gold filling. We cannot think that the instances are parallel. Our correspondent confounds ready cohesion with the ordinary process of welding. When two cohesive surfaces are brought into absolute contact, they unite without either pressure or a blow. If two pieces of soft wax are brought together, they cohere at once. If two pieces of iron are heated almost to the melting point, their particles unite upon being brought together, because cohesion is nearly destroyed and the particles intermingle, as in the case of colloids, and then recrystallize. But iron at a lower temperature is not cohesive, and it is with great difficulty that the molecules of two different pieces are made to unite.

This in not the case with gold—at least not to the same degree. If two surfaces of perfectly pure gold, made entirely clean, are put in absolute contact, they at once cohere, and no blow is necessary for their union. Theoretically then, gold should be easily welded by mere pressing or rubbing the surfaces together. But practically it is very difficult to obtain gold that is in the proper condition. It is almost impossible to keep foil absolutely clean. There will be a film deposited upon its surface, from the atmosphere or from the leaves of the book in which it is contained, and this will be fatal to ready coherence. A blow upon a serrated plugger will cause an interlacing of the particles, and will break up the film upon the surface and thus bring the particles into immediate contact, allowing them to unite at once. If we heat the gold we drive off this film, and it becomes "cohesive" or "adhesive," but the pieces now unite so readily that "bridging" is liable to occur. To our apprehension, the gold that is in a fit condition to readily unite under the rotary system is impracticable on this account, and hence we choose that which is less clean, and "soft" enough to permit the sliding of one surface upon another, and then resort to the mallet to secure the necessary consolidation.

